# Case of Partial Perspiration

**Published:** 1814-05

**Authors:** 


*For the Medical and Physical Journal.*

*Case of partial Perspiration.*

I SHALL esteem it a favour if you will insert the following case in your Journal.—A neighbour of mine has something in his constitution different from what I have ever met with. When he works he generally sweats profusely on the right side; it takes one half of his face and of his body, so that you may see the sweat pouring down in large drops, while at the same time his left side is cool and dry; and when he eats the perspiration changes to the left side, with as great a profusion as before, while the right side is perfectly dry. Thus by working and eating it alternately changes from right to left and left to right, but he never perspires all over his face and body at the same time. If any of your correspondents can account for this phenomenon in nature, it will much oblige A. C. R.

